# Assessing the relationship between transformational leadership and organizational commitment in health sciences colleges: a mixed-methods study

**DOI:** 10.3389/fmed.2026.1865356

**Published:** 2026-08-05

**Authors:** Zeinab A. Abusabeib, Yosra A. Ali

**Affiliations:** 1 Department of Community and Psychiatric Mental Health Nursing, College of Nursing, Princess Nourah Bint Abdulrahman University, Riyadh, Saudi Arabia; 2 Pediatric Dentistry Department, National Ribat University, Khartoum, Sudan

**Keywords:** health sciences education, leadership, organizational commitment, Saudi Arabia, transformational leadership

## Abstract

**Background:**

Transformational leadership has been recognized as a critical factor influencing employee commitment in educational environments, but research exploring this connection within higher education in Saudi Arabia remains limited. The study aimed to assess the relationship between transformational leadership and organizational commitment among faculty members in academic leadership roles in the Colleges of Health Sciences in Saudi Arabia.

**Methods:**

A mixed-methods cross-sectional design was employed, and quantitative data were collected from 155 faculty members in academic leadership roles using a total population approach. Transformational leadership was measured using the Multifactor Leadership Questionnaire, while organizational commitment was assessed using Meyer and Allen's three-component model of organizational commitment. Statistical analysis included descriptive statistics, reliability analysis, normality assessment, Pearson's correlation, and multiple linear regression. In addition, focus group discussions were conducted to explore participants' perceptions of leadership practices and their influence on organizational commitment within health sciences education settings.

**Results:**

Transformational leadership was perceived at a relatively high level, with inspirational motivation being the most prominent dimension. Inspirational motivation showed the strongest correlation with affective commitment (*r* = 0.63, *p* < 0.01). Regression analysis indicated that inspirational motivation and idealized influence significantly predicted affective commitment, whereas idealized influence was the primary predictor of normative and continuance commitment. Qualitative findings supported these results, highlighting the importance of visionary leadership, role modeling, recognition, and supportive leadership practices in strengthening organizational commitment.

**Conclusion:**

The results suggest that transformational leadership is essential for fostering educators' emotional and normative commitment in health professions education. By promoting vision, mentorship, and appreciation, academic leaders can increase faculty engagement and loyalty to the institution. The research highlights the importance of leadership development programs as part of ongoing professional development strategies to sustain faculty motivation and enhance educational quality within health sciences institutions.

## Introduction

Leadership plays a crucial role in shaping the direction, performance, and culture of an organization, particularly in education, where it is directly associated with both faculty engagement and student outcomes (1–3). In higher education institutions, leadership significantly affects teaching outcomes, faculty retention, institutional culture, and the long-term sustainability of the organization. Among the various leadership models, transformational leadership has gained attention for its emphasis on inspiring and motivating individuals to rise above their personal interests and commit to collective organizational objectives. In transformational leadership, shared decision-making is emphasized, and power is exercised through facilitation, consensus, and not over others (4). This leadership style is particularly significant in the context of Saudi Arabia's Vision 2030 reform agenda, which prioritizes institutional performance, educational quality, and innovation in public higher education. As institutions of higher education adapt to globalization, innovation, and national development initiatives, effective leadership has become vital for organizational success. Transformational leadership is recognized for its capacity to motivate faculty, shape organizational culture, and improve educator engagement (3, 4).

Transformational leadership includes four essential dimensions: idealized influence, inspirational motivation, intellectual stimulation, and individualized consideration. Leaders who demonstrate idealized influence act as role models, cultivating trust and admiration among followers through their integrity and common values. As a result of idealized influence behavior, work is accomplished based on a cooperative sagacity of values, purpose, and belief (4–7). Inspirational motivation involves articulating a compelling vision that inspires and aligns people with the organization's goals. As a result, followers can recognize the shared vision and express it to others (1, 7). Intellectual stimulation promotes creativity and effective problem-solving by questioning traditional methods, while individualized consideration focuses on personal growth and meeting the distinct needs of team members (4–8). These skills enable leaders to assist their members in becoming fully actualized individuals by understanding and addressing their needs (1). According to Bass and Avolio's model, transformational leaders also “Model the Way,” “Inspire a Shared Vision,” “Challenge the Process,” “Enable Others to Act,” and “Encourage the Heart,” which fosters collaboration, innovation, and stakeholder engagement (3).

In higher education institutions, including health sciences colleges, transformational leadership has been shown to enhance organizational commitment, teacher satisfaction, and overall institutional effectiveness. A commitment to an organization can be defined as a desire to remain a member, a desire to work hard in accordance with the organization's wishes, and acceptance of the organization's values and goals (9). Organizational commitment is a complex concept that includes affective, continuance, and normative commitment. Affective commitment refers to the emotional connection to the organization, continuance commitment is based on the perceived costs associated with leaving, and normative commitment is driven by a sense of moral duty. When commitment is effective, job performance improves, job satisfaction improves, leadership skills develop, and turnover is reduced (10). Research consistently indicates that transformational leadership has a positive effect on organizational commitment by fostering a supportive, inclusive, and motivating atmosphere (11, 12). According to Kreitner and Kinick (13), transformational leadership enhances the commitment of both individuals and organizations through its impact on shared values and motivation.

Studies consistently show that transformational leadership has a positive effect on all these aspects by fostering a supportive, inclusive, and motivating atmosphere (14). However, educational systems are still confronted with issues such as inadequately trained teachers, insufficient empowerment, and unfavorable working conditions, which diminish teacher dedication. Leadership approaches that focus on vision, mentorship, and innovation, such as transformational leadership, have the potential to tackle these problems (5, 15). Nevertheless, there is limited knowledge regarding the extent to which this leadership style is applied within Saudi health sciences colleges and its impact on faculty engagement and organizational commitment.

Although transformational leadership has been widely studied internationally, there is limited empirical evidence on its relationship with organizational commitment in Saudi health science colleges. Although international studies demonstrate a positive correlation between transformational leadership and employee commitment, there is limited research specifically focused on Saudi Arabia, particularly in the health sciences field (16, 17). This raises questions about how transformational leadership functions within structured and culturally specific academic settings and whether hybrid or transactional leadership styles might occasionally complement transformational approaches in health sciences colleges (5, 18–20).

The current study is conceptually grounded in transformational leadership theory (20) and Meyer and Allen's three-component model of organizational commitment. Transformational leadership theory posits that value-based leadership behaviors, such as inspirational motivation and idealized influence, shape followers' emotional attachment and identification with organizational goals within the academic environment. Concurrently, the three-component model distinguishes among affective, normative, and continuance commitment, enabling a nuanced analysis of how leadership behaviors may differentially influence these dimensions. Drawing upon transformational leadership theory and Meyer and Allen's three-component model of organizational commitment, this study examines the following hypotheses:

H1: Transformational leadership practices are positively associated with affective commitment among faculty members holding academic leadership positions.

H2: Transformational leadership practices are positively associated with normative commitment among faculty members holding academic leadership positions.

H3: Transformational leadership practices are positively associated with continuance commitment among faculty members holding academic leadership positions; however, this association is anticipated to be weaker than that with affective and normative commitment.

H4: Among the dimensions of transformational leadership, inspirational motivation and idealized influence are anticipated to demonstrate the strongest associations with affective commitment (10, 20).

Based on this theoretical foundation, this study seeks to address that gap by investigating how leadership practices can be tailored to align with cultural and institutional needs. This study aimed to investigate the relationship between transformational leadership practices and organizational commitment among faculty members holding academic leadership positions in Saudi health sciences colleges. The objectives were to: (1) assess the perceived level of transformational leadership practices; (2) determine the levels of affective, continuance, and normative commitment; (3) examine the associations between transformational leadership dimensions and organizational commitment dimensions; (4) identify which transformational leadership dimensions independently predict organizational commitment; and (5) explore faculty members' perceptions regarding the influence of leadership practices on their organizational commitment within the context of Saudi health sciences education.

The study employs the Multifactor Leadership Questionnaire (MLQ-6S) and the Organizational Commitment Model by Meyer and Allen (10, 20, 21), integrating quantitative analysis with qualitative insights gathered from focus group discussions (FGDs) to capture the contextual dynamics of Saudi higher education. The outcomes aim to guide leadership development efforts, enhance faculty involvement, and support the overarching objectives of educational reform in Saudi Arabia.

## Materials and methods

### Study design

An explanatory sequential mixed-methods cross-sectional design was utilized in this study. This research design is suitable when qualitative findings are employed to explain or provide context for initial quantitative results (22, 23). The initial phase involved collecting quantitative survey data to assess transformational leadership practices, dimensions of organizational commitment, and the statistical relationships among these variables. Subsequently, qualitative data were gathered through focus group discussions to explain and contextualize the quantitative results. These qualitative findings offered a deeper interpretation of the ways in which leadership practices affect faculty members' emotional, normative, and continuance commitment within Saudi health sciences education.

### Study area/setting

The study was conducted in an educational institution in Saudi Arabia. Specifically, the research focused on the Colleges of Health Sciences, providing both undergraduate and postgraduate degrees. Faculty members in academic leadership roles in this setting are actively involved in teaching, clinical supervision, research, and academic leadership.

### Variables under study

Independent variables were transformational leadership practices, assessed through the MLQ-6S, including Idealized attributes, inspirational motivation, intellectual stimulation, and individualized consideration.

Dependent variables include components of the Organizational Commitment Model, namely affective commitment, continuance commitment, and normative commitment.

Other variables include demographic characteristics such as age, academic rank, years of experience, and department affiliation.

### Study population

The intended target population consisted of faculty members in academic leadership roles, including professors, associate professors, assistant professors, and lecturers, at the Colleges of Health Sciences, which encompass the faculties of Medicine, Dentistry, Pharmacy, Nursing, Health Sciences, and Rehabilitation.

The total number of leaders across all health colleges was 183. Of these, 155 respondents participated in the study, yielding a response rate of 84.7%.

### Sampling method and sample size

A total population approach was employed. All eligible faculty members in academic leadership roles across the Colleges of Health Sciences were invited to participate. Of the 183 eligible faculty leaders, 155 completed the quantitative survey, resulting in a response rate of 84.7%. Consequently, the study did not utilize probabilistic sampling but instead sought to include the entire accessible population of faculty leaders within the participating institution.

For the qualitative component, participants were chosen from quantitative survey respondents who expressed willingness to participate in follow-up FGDs, with each group comprising 5–7 faculty members selected from the original pool of respondents. Selection was designed to maximize variation in academic rank, college affiliation, and years of experience where feasible.

### Inclusion and exclusion criteria

Inclusion criteria: Faculty members who have had a minimum of 1 year of experience at the college and are presently engaged in teaching and academic leadership.

Exclusion criteria: Faculty members on long-term leave or those with less than 1 year of service. Those who refused to participate were also excluded.

### Data collection techniques

#### Quantitative data collection

A self-administered online questionnaire was distributed via Google Forms to various participants, incorporating the MLQ-6S and the commitment model, following an explanation of the study's purpose, content, and duration.

The MLQ-6S was used to assess transformational leadership (including idealized influence, inspirational motivation, intellectual stimulation, and individualized consideration), as well as transactional and laissez-faire leadership styles. It contains 21 items that are rated using a 5-point Likert scale (24).

MLQ was developed by Bass to measure transformational leadership constructs and other domains of leadership theory, such as transactional (active, passive, and contingent reward) and laissez-faire leadership. In this study, the Multifactor Leadership Questionnaire, form 6-S (MLQ-6S) was used (24). A wide range of organizational settings are used to apply this instrument, which is referred to as the most frequently validated and well-researched leadership instrument in the world (20, 21).

Its reliability has been verified using several different approaches, including examining the agreement among respondents (20). Testing and retesting have confirmed its reliability when examining leadership indicators (25). It has been reported that Cronbach's alpha coefficient for the MLQ ranges from 0.74 to 0.94, which is considered acceptable and reliable by the authors (26).

Several studies have demonstrated that the MLQ is a valid instrument across various validity types, including those conducted by Tejeda, Avolio, and Bass. According to Tejeda, a reduced set of items from the questionnaire showed predictive and construct validity. Secondly, the convergent validity was supported by a high degree of intercorrelation of the transformational items. Thirdly, the transformational leadership scales showed adverse relations with both laissez-faire leadership and management by exception subscales, suggesting discriminant validity (21, 27).

Meyer and Allen's organizational commitment model was used to evaluate affective, continuance, and normative commitment. It consists of 24 items categorized into three dimensions: desire-based commitment (i.e., affective commitment), obligation-based commitment (i.e., normative commitment), and cost-based commitment (i.e., continuance commitment) (10).

Both tools were shared electronically through email, with prior consent received from all participants. Participants were directed to respond autonomously and confidentially. The data collection took place over a 4-week period.

A quantitative cross-analysis of the MLQ-6S findings and Organizational Commitment Questionnaire responses was conducted to evaluate the relationships between leadership approach and commitment and to determine which leadership style is most strongly associated with higher commitment. The instruments were administered in English, as it is the primary academic language in health sciences colleges in Saudi Arabia. No structural modifications were made to the validated scales.

#### Qualitative data collection

A semi-structured FGD was conducted with five groups, each comprising 5–7 participants. The session was conducted online via Zoom and was facilitated by the principal researcher. An interview guide was used to maintain consistency across themes, focusing on participants' perceptions of leadership practices and their impact on commitment. The focus group guide was developed after preliminary review of the quantitative findings, particularly the strong association between inspirational motivation, idealized influence, and affective commitment, and the weaker associations observed for continuance commitment. The discussion lasted around 60 min and was audio-recorded with the participants' permission.

The qualitative sample included 32 participants across the five FGDs. Recruitment continued until the planned focus groups were completed, and no substantially new themes were identified.

### Data processing and analysis

Quantitative data were entered into and analyzed using the Statistical Package for Social Sciences (SPSS) version 26. Descriptive statistics, including frequencies, percentages, means, and standard deviations, were utilized to describe demographic characteristics and key variables. Prior to conducting inferential analyses, the normality assumption was assessed using the Shapiro–Wilk test and skewness and kurtosis values. Pearson's correlation coefficient was used to explore the relationship between components of transformational leadership and dimensions of organizational commitment.

Multiple linear regression analyses were conducted to more rigorously assess the predictive relationships between transformational leadership and organizational commitment. Three separate regression models were specified, each using affective commitment, continuance commitment, or normative commitment as the dependent variable. The four dimensions of transformational leadership—idealized influence, inspirational motivation, intellectual stimulation, and individualized consideration—served as independent variables. Multicollinearity was evaluated using variance inflation factor values. Internal consistency reliability was assessed using Cronbach's alpha coefficient for the overall questionnaire, the two main scales, and their respective subscales. As all quantitative variables were collected through self-reported questionnaires at a single time point, the potential for common method bias was taken into account when interpreting the findings.

Qualitative data from the focus group discussions (FGDs) were transcribed verbatim and analyzed using thematic analysis with NVivo software. This analysis employed an inductive approach, which included repeated reading of transcripts, initial coding, grouping related codes into categories, and developing overarching themes. Integration of qualitative and quantitative findings occurred during interpretation by comparing statistical patterns with themes identified from the FGDs.

This study followed the STROBE (Strengthening the Reporting of Observational Studies in Epidemiology) reporting guidelines.

A qualified biostatistician reviewed all statistical procedures to ensure methodological rigor and accurate interpretation of results.

### Ethical consideration

Ethical approval was obtained from the Institutional Review Board (IRB) of the participating colleges. All participants were informed about the study's objectives, the voluntary aspect of their involvement, and the measures in place to ensure confidentiality. Consent was acquired electronically prior to their participation. Anonymity was assured, and participants had the right to withdraw at any stage.

All data were securely stored in password-protected files accessible solely to the researchers. The study followed ethical guidelines in accordance with the Declaration of Helsinki.

## Results

### Demographic characteristics of respondents

The study included 155 faculty members in academic leadership roles from Colleges of Health Sciences in Saudi Arabia, with an 84.7% response rate. Participants varied in terms of age, academic rank, and years of professional experience, providing a diverse representation of faculty members holding academic leadership roles within health sciences institutions. Detailed demographic characteristics of the respondents are presented in Table 1.

**Table 1:** Demographic characteristics of respondents (*n* = 155).

Characteristic	Frequency (*n*)	Percentage (%)
Age (years)		
20–29	0	0.0
30–39	56	36.1
40–49	65	41.9
50–59	31	20.0
Academic position		
Professor	2	1.3
Associate professor	10	6.5
Assistant professor	128	82.6
Lecturer	15	9.7

### Assessment of normality

Normality assessment indicated that the primary study variables were approximately normally distributed. The Shapiro–Wilk tests were non-significant for all dimensions of transformational leadership and organizational commitment, with *p-values* ranging from 0.127 to 0.663. Skewness ranged from −0.345 to 0.103, and kurtosis ranged from −0.355 to 0.950, supporting the application of Pearson's correlation and multiple linear regression analyses.

### Reliability analysis

The Cronbach's alpha coefficients revealed that the study measures had acceptable to excellent internal consistency. The overall questionnaire showed excellent reliability (α = 0.91). The transformational leadership scale displayed strong internal consistency (α = 0.92), with subscale alphas ranging from 0.84 to 0.88. Specifically, idealized influence had a Cronbach's alpha of 0.86, inspirational motivation 0.88, intellectual stimulation 0.84, and individualized consideration 0.85. The organizational commitment scale also had good internal consistency (α = 0.88), and its subscales demonstrated acceptable reliability: affective commitment (α = 0.87), continuance commitment (α = 0.76), and normative commitment (α = 0.82).

### Prevalence of transformational leadership practices

Table 2 presents the mean scores for the transformational leadership components on the MLQ-6S. Transformational leadership practices were rated highly, with Inspirational Motivation achieving the highest score (mean = 3.44), followed closely by Idealized Influence (3.38). Intellectual Stimulation and Individualized Consideration had slightly lower but still positive mean scores.

**Table 2:** Mean scores of transformational leadership components.

Dimension	Mean	SD
Idealized influence	3.38	0.45
Inspirational motivation	3.44	0.45
Intellectual stimulation	3.29	0.45
Individualized consideration	3.25	0.45

### Levels of organizational commitment

Table 3 indicates the mean scores for the three dimensions of organizational commitment: affective, continuance, and normative. The highest mean score was observed in Affective Commitment (3.41), indicating a relatively stronger emotional attachment to the institutions, followed by Normative Commitment (3.31) and Continuance Commitment (3.22).

**Table 3:** Mean scores of organizational commitment dimensions.

Commitment dimension	Mean	SD
Affective commitment	3.41	0.59
Continuance commitment	3.22	0.61
Normative commitment	3.31	0.64

### Correlation between leadership and commitment

Pearson correlation analysis revealed significant positive relationships between most transformational leadership components and the three dimensions of organizational commitment. Table 4 shows the correlation coefficients between transformational leadership components and organizational commitment dimensions. The strongest correlation was found between Inspirational Motivation and Affective Commitment (*r* = 0.63, *p* < 0.01), followed by Idealized Influence and Affective Commitment (*r* = 0.61, *p* < 0.01). Correlations with normative commitment were moderate (*r* = 0.34–0.45, *p* < 0.01), and correlations with continuance commitment were weaker. The weakest correlation was observed between intellectual stimulation and continuance commitment, although this association remained statistically significant (*r* = 0.19, *p* = 0.018).

**Table 4:** Correlation between transformational leadership and organizational commitment.

Leadership component	Affective commitment	Continuance commitment	Normative commitment
Idealized influence	0.61 (*p* < 0.01)	0.32 (*p* < 0.05)	0.45 (*p* < 0.01)
Inspirational motivation	0.63 (*p* < 0.01)	0.28 (*p* < 0.05)	0.39 (*p* < 0.01)
Intellectual stimulation	0.47 (*p* < 0.01)	0.19 (*p* = 0.018)	0.36 (*p* < 0.01)
Individualized consideration	0.50 (*p* < 0.01)	0.23 (*p* < 0.05)	0.34 (*p* < 0.01)

### Multiple regression analysis

Multiple linear regression analyses were performed to determine which transformational leadership dimensions independently predicted each organizational commitment dimension. The regression model for affective commitment was statistically significant and accounted for 45.6% of the variance, with inspirational motivation and idealized influence identified as significant predictors. The model for continuance commitment was also statistically significant, but explained a smaller proportion of variance, with idealized influence as the sole significant predictor. Similarly, the model for normative commitment was statistically significant, accounting for 22.2% of the variance, with idealized influence remaining the only significant independent predictor. No serious multicollinearity was observed, as variance inflation factor values were within acceptable limits. Complete regression coefficients are provided in Table 5.

**Table 5:** Multiple linear regression models predicting organizational commitment dimensions.

Outcome	Predictor	*B*	SE	β	*p*-value
Affective commitment	Idealized influence	0.350	0.125	0.267	0.006
Affective commitment	Inspirational motivation	0.456	0.121	0.348	< 0.001
Affective commitment	Intellectual stimulation	0.073	0.105	0.056	0.489
Affective commitment	Individualized consideration	0.125	0.108	0.095	0.248
Continuance commitment	Idealized influence	0.330	0.165	0.244	0.047
Continuance commitment	Inspirational motivation	0.142	0.160	0.105	0.377
Continuance commitment	Intellectual stimulation	−0.050	0.139	−0.037	0.722
Continuance commitment	Individualized consideration	0.047	0.142	0.035	0.741
Normative commitment	Idealized influence	0.416	0.162	0.292	0.011
Normative commitment	Inspirational motivation	0.131	0.157	0.092	0.407
Normative commitment	Intellectual stimulation	0.152	0.136	0.107	0.268
Normative commitment	Individualized consideration	0.065	0.139	0.046	0.642

B, unstandardized regression coefficient; SE, standard error; β, standardized regression coefficient. VIF values ranged from 1.77 to 2.49, indicating no serious multicollinearity.

Model statistics were as follows: affective commitment, *F*_(4, 150)_ = 31.37, *p* < 0.001, R^2^ = 0.456, adjusted R^2^ = 0.441; continuance commitment, *F*_(4, 150)_ = 4.54, *p* = 0.002, R^2^ = 0.108, adjusted R^2^ = 0.084; and normative commitment, *F*_(4, 150)_ = 10.68, *p* < 0.001, R^2^ = 0.222, adjusted R^2^ = 0.201.

### Focus group results (qualitative)

Analysis of the five FGDs yielded the following themes related to leadership experiences and organizational commitment.

Inspirational Leadership Enhances Engagement and shared purpose: Faculty valued leaders who clearly communicated a vision and motivated staff. “When our dean shares the vision passionately, we feel inspired and united,” noted one participant.

Role modeling builds trust: Ethical and approachable leaders were perceived as more effective.

Gaps in Individualized Mentorship: Several faculty members highlighted gaps in individualized mentorship, particularly for junior academics.

Recognition strengthens commitment: Faculty loyalty was strongly linked to leaders' recognition of their contributions.

Mixed views on other leadership styles: While transformational leadership was preferred, some transactional practices were considered necessary for operational control.

### Integration of quantitative and qualitative findings

Integration of quantitative and qualitative findings demonstrated convergence between statistical results and participants' narratives. Quantitative analysis indicated that inspirational motivation and idealized influence exhibited the strongest relationships with affective commitment and were significant predictors in the regression model. Qualitative data revealed that visionary communication, ethical role modeling, recognition, and trust-building enhanced emotional attachment to the institution. The weaker association between transformational leadership and continuance commitment aligned with participants' perspectives that continuance commitment is shaped more by structural factors such as workload, job security, financial considerations, and career opportunities than by leadership behaviors alone. These integrated findings indicate that transformational leadership primarily enhances emotional and value-based forms of commitment, whereas continuance commitment is more strongly influenced by institutional policies and employment conditions (23, 28).

## Discussion

### Principal findings and comparison with prior work

This study examined the relationship between transformational leadership and faculty members' organizational commitment (affective, normative, and continuance) in academic leadership roles at health sciences colleges in Saudi Arabia. These findings are particularly relevant in health sciences education settings, where faculty members often balance teaching, clinical supervision, research, and academic leadership responsibilities.

The sample was predominantly mid-career, with Assistant Professors forming the overwhelming majority (82.6%), followed by Lecturers (9.7%), Associate Professors (6.5%), and Professors (1.3%). These demographics are relevant when interpreting how inspirational and role-model behaviors strengthened affective commitment among faculty in this study.

Transformational leadership was perceived as being practiced with moderate to high frequency, with Inspirational Motivation and Idealized Influence standing out as the most significant dimensions of transformational leadership. This suggests that leaders were strongly perceived as visionaries and role models, with opportunities to further enhance individualized mentorship.

When compared with previous studies, these results align with international evidence showing that transformational leadership is widely practiced globally and strongly fosters emotional attachment and loyalty. These findings are also consistent with Saudi-based evidence showing that leadership practices are associated with organizational commitment in healthcare and higher education contexts (16, 17, 29). Studies in higher education and healthcare sectors globally, such as those in South Africa and Ethiopia, highlight the role of Inspirational Motivation and Idealized Influence in cultivating a strong sense of purpose and identity among faculty members (5, 18, 19, 30).

The correlational analysis revealed the strongest associations between transformational leadership components and affective commitment, specifically Inspirational Motivation and Idealized Influence. These findings indicate that leadership behaviors associated with vision and role modeling are especially important for enhancing emotional attachment to the institution.

Thematic analysis supported this connection: faculty consistently noted that leaders who articulate a compelling vision, exemplify the values they advocate, and publicly acknowledge staff enhance emotional loyalty and pride in the workplace.

These findings are closely aligned with an extensive array of educational and organizational studies that demonstrate that transformational leaders increase followers' emotional attachment to their organizations (12, 31). Several studies reported similar patterns across various contexts—greater affective commitment when transformational behaviors were assessed positively (such as in Malaysian and Ethiopian schools and universities, and among nursing samples in India). Studies employing MLQ instruments and commitment scales commonly identify inspirational communication and role modeling as key drivers of affective bond formation. The qualitative focus on leader authenticity, recognition, and a shared vision resonates with insights from comparative research in higher education and healthcare (5, 18, 19, 30).

Transformational leadership theory suggests three mechanisms that may explain the observed associations. Emotional contagion and meaning making: Inspirational leaders foster a sense of purpose and collective identity; when work is perceived as meaningful, affective attachment deepens.

Trust and identification: Idealized Influence fosters trust; when staff aligns with leaders' values, they embrace organizational objectives.

Recognition and psychological support: Praise, visibility, and encouragement for career advancement (elements of individualized consideration and contingent rewards) enhance the relationship.

Given the demographic characteristics, with the majority in mid-career (aged 40–49) and a significant proportion being assistant professors, these mechanisms hold particular importance. Mid-career faculty typically pursue recognition, growth opportunities, and alignment with institutional missions—areas that inspirational and exemplary leaders fulfill.

The components of transformational leadership also exhibited a positive correlation with normative commitment, indicating that transformational behaviors were associated with faculty members' feelings of obligation and moral responsibility toward their institutions, albeit not as strongly as with affective commitment.

The established positive relationship between transformational leadership and normative commitment is supported by numerous studies (5, 19, 32), indicating that transformational leaders who articulate values and foster a culture of shared purpose often enhance employees' sense of moral obligation to remain with the organization.

Some comparative research shows that normative commitment is more influenced by context—for example, in collectivist societies or institutions with strong professional norms, normative commitment might be relatively high. The reasons behind this relationship are likely to include mechanisms such as value alignment and internalized norms. Idealized Influence and Inspirational Motivation promote a match between personal values and organizational norms; when employees sense this alignment, they may feel a moral obligation to reciprocate with loyalty. Furthermore, socialization and organizational practices: Leadership styles that prioritize long-term dedication and institutional pride can nurture normative responsibilities (e.g., celebrating milestones and formal commitment declarations). In the context of Saudi Arabia, which is both collegial and hierarchical, characterized by strong institutional expectations, the effects of normative commitment may be heightened: leaders who embody institutional values can evoke a sense of duty that extends beyond personal preference.

The correlation between transformational leadership and continuance commitment was found to be less strong compared to affective or normative commitment. The empirical findings on transformational leadership and continuance commitment are inconsistent in the literature. Some studies (5, 18, 19) report positive relationships between transformational leadership and continuance commitment, while others report minimal or varying effects. School-based studies have occasionally identified continuance as the predominant commitment type when job stability and benefits were significant. The findings of this study—a modest transformational leadership –continuance link—align with the perspective that continuance commitment is primarily based on costs and is less influenced by leadership behaviors than affective or normative commitment.

Possible reasons for this relationship include:

Cost/benefit perspective: Continuance commitment is influenced by the perceived costs of leaving (such as financial implications, pensions, and limited external job opportunities). Transformational leadership typically has a greater impact on emotional and value-based connections than on financial considerations.

Organizational policies: Factors such as compensation, contract terms, labor market alternatives, and career opportunities likely exert greater influence on continuance commitment than leadership styles do.

Indirect effects: Transformational leadership may affect continuance commitment indirectly, for example, by enhancing opportunities for development or advancement (thus increasing the perceived value of remaining), but these pathways were not specifically assessed in this analysis.

Qualitative insights gathered from five FGDs supported the quantitative findings: highlighting the positive impact of inspirational, role-model leadership on morale and loyalty, while junior faculty reported gaps in individualized mentoring.

These patterns provide a framework for interpretation: transformational leadership, particularly the inspirational and role-modeling elements, most significantly fosters emotional attachment (affective commitment). Transformational leadership has a notable but limited influence on normative (obligation-based) commitment and a modest effect on continuance (cost-based) commitment, the latter of which appears to be driven primarily by structural and economic considerations.

These findings align with Leithwood and Jantzi's (33) observation that transformational leadership is associated with higher levels of commitment, but its success is contingent upon contextual factors, such as the availability of mentoring systems, faculty development opportunities, and institutional support structures. The emphasis on recognition and trust-building found in this study is consistent with findings in other Gulf and Asian contexts, where collectivist cultural norms place a high value on respect and interpersonal relationships between leaders and faculty.

These findings provide insights for leadership development and institutional governance within higher education systems undergoing reform, particularly in the context of ongoing educational transformation in Saudi Arabia.

### Limitations

This study presents several limitations. First, its cross-sectional and correlational design prevents determining a causal relationship between transformational leadership and organizational commitment. Second, while a total population approach was applied within the institution, the focus on a single institution and the predominance of assistant professors may restrict the applicability of results to other universities, regions, or senior academic leadership groups. The very small number of professors (*n* = 2) also constrained subgroup analysis and may not adequately reflect senior leadership views. Third, all quantitative data were collected via self-reported questionnaires at one point in time, potentially introducing common method bias and social desirability effects. Fourth, factors relevant to continuance commitment—such as salary, contract type, nationality, promotion opportunities, and external labor market conditions—were not assessed. Finally, although qualitative focus groups provided deeper context for interpreting the quantitative findings, all participants were from the same institution; future research should incorporate broader, multi-institutional samples.

## Conclusions

This research provides new insights into the relationship between transformational leadership and faculty leaders' organizational commitment in the Colleges of Health Sciences in Saudi Arabia. The findings suggest that transformational leadership is practiced at a relatively high level and was positively associated with both affective and normative commitment, suggesting that faculty members feel emotionally connected and morally aligned with the institution's values when leaders exhibit vision, integrity, and recognition. In contrast, continuance commitment showed weaker associations with leadership style, highlighting the role of structural and economic factors in faculty retention. These results underscore the relevance of transformational leadership in higher education, providing culturally grounded evidence for incorporating this leadership style into development strategies within Saudi institutions.

The qualitative findings underscore the importance of mentorship, recognition, role modeling, and personalized support, suggesting that future leadership programs should integrate visionary leadership training with practical systems to address faculty development and retention challenges. By aligning leadership practices with the objectives of Vision 2030, health sciences institutions may be better positioned to enhance faculty engagement and institutional loyalty within an evolving educational environment.

This research enriches the existing literature by addressing a gap in the literature on transformational leadership in Middle Eastern higher education, presenting practical suggestions for leadership training, faculty support mechanisms, and retention strategies. The findings lay an evidence-based groundwork for future studies and strategic initiatives, emphasizing the need to integrate leadership development with institutional culture, organizational policies, and national reform efforts.

Additionally, this study highlights the importance of employing validated international tools, such as the MLQ-6S and the Organizational Commitment Model, in a Middle Eastern context, thereby enhancing cross-cultural leadership research. Merging quantitative findings with qualitative feedback from faculty focus groups provides a comprehensive understanding of leadership effectiveness and its direct impact on faculty engagement and institutional outcomes.

The research recommends implementing structured leadership development programs to enhance transformational leadership behaviors, ethical role modeling, and mentoring skills through workshops, coaching, and 360-degree feedback. Creating consistent recognition programs and effective feedback systems can reinforce positive behaviors and enhance commitment. Workplace wellbeing initiatives that tackle workload concerns, promote work-life balance, and support faculty mental health are also crucial. Strategic objectives, such as alignment with Vision 2030 priorities, must be translated into practical support mechanisms, including seed grants and workload distribution, to ensure that inspirational messaging yields tangible benefits for faculty. At the policy level, institutions should incorporate transformational leadership competencies into the criteria for selecting and assessing leadership performance, and develop retention strategies that emphasize intrinsic motivators, including professional development and academic autonomy, alongside extrinsic rewards.

For future research, it is advisable to obtain a broader sample across varied regions and higher education sectors to improve generalizability, alongside conducting longitudinal studies to explore the long-term effects of transformational leadership on faculty commitment and employing mixed-method approaches that integrate qualitative and quantitative data for a deeper understanding of how transformational leadership behaviors impact commitment dynamics.

These findings support the value of leadership development initiatives that emphasize vision, recognition, and individualized faculty support. Overall, the study highlights the importance of leadership development in enhancing organizational commitment and strengthening academic environments within health sciences education settings undergoing reform in Saudi Arabia.

## Data Availability

The raw data supporting the conclusions of this article will be made available by the authors, without undue reservation.
